# *Zea mays* RNA-seq estimated transcript abundances are strongly affected by read mapping bias

**DOI:** 10.1186/s12864-021-07577-3

**Published:** 2021-04-20

**Authors:** Shuhua Zhan, Cortland Griswold, Lewis Lukens

**Affiliations:** 1 Department of Plant Agriculture, University of Guelph, Guelph, Ontario Canada; 2 Department of Integrative Biology, University of Guelph, Guelph, Ontario Canada

**Keywords:** Mapping bias, eQTL analysis, Sequence divergence, Gene coexpression analysis, Maize, RNA-Seq, Genetic diversity, Transcriptome variation

## Abstract

**Background:**

Genetic variation for gene expression is a source of phenotypic variation for natural and agricultural species. The common approach to map and to quantify gene expression from genetically distinct individuals is to assign their RNA-seq reads to a single reference genome. However, RNA-seq reads from alleles dissimilar to this reference genome may fail to map correctly, causing transcript levels to be underestimated. Presently, the extent of this mapping problem is not clear, particularly in highly diverse species. We investigated if mapping bias occurred and if chromosomal features associated with mapping bias. *Zea mays* presents a model species to assess these questions, given it has genotypically distinct and well-studied genetic lines.

**Results:**

In *Zea mays*, the inbred B73 genome is the standard reference genome and template for RNA-seq read assignments. In the absence of mapping bias, B73 and a second inbred line, Mo17, would each have an approximately equal number of regulatory alleles that increase gene expression. Remarkably, Mo17 had 2–4 times fewer such positively acting alleles than did B73 when RNA-seq reads were aligned to the B73 reference genome. Reciprocally, over one-half of the B73 alleles that increased gene expression were not detected when reads were aligned to the Mo17 genome template. Genes at dissimilar chromosomal ends were strongly affected by mapping bias, and genes at more similar pericentromeric regions were less affected. Biased transcript estimates were higher in untranslated regions and lower in splice junctions. Bias occurred across software and alignment parameters.

**Conclusions:**

Mapping bias very strongly affects gene transcript abundance estimates in maize, and bias varies across chromosomal features. Individual genome or transcriptome templates are likely necessary for accurate transcript estimation across genetically variable individuals in maize and other species.

**Supplementary Information:**

The online version contains supplementary material available at 10.1186/s12864-021-07577-3.

## Background

Gene expression differences among genetically distinct individuals contribute to phenotypic differences. For example, disease resistant genotypes in plants may suppress the expression of genes that cause them to be susceptible to a pathogen [1] or have distinct transcriptome responses to pathogens [2]. In response to abiotic stresses, distinct transcripts underlie genotypes with different secondary metabolite accumulation and survival [3, 4]. Developmentally, flowering time [5], seed size [6, 7], and fruit size [8] all vary because of gene regulatory variation. Selection may act on these regulatory differences [9].

RNA-seq is the major platform for genome-wide assessment of transcript abundances [10]. A standard practice is to first assign RNA-seq reads to a single, high quality reference genome or transcriptome and second to use the number of assigned reads as an indicator of transcript abundance [10, 11]. Nonetheless, estimating gene expression using this method across genetically distinct individuals can be problematic. An RNA-seq read may map to its source gene, but an RNA-seq read from an allele dissimilar to the reference allele may not map [12] or may map to an incorrect genomic location [13]. Preferential alignment of reads can be detected if transcript levels from different genotypes are systematically unequal.

The extent to which genetic variation affects RNA-seq based transcript estimates correlates with genetic distance. Quinn et al. (2014) aligned RNA-seq reads from the F1 progeny of a cross between two inbred *Drosophila melanogaster* lines to the *D. melanogaster* reference genome and estimated allele transcript abundances at polymorphic sites. The expected proportion of aligned reads carrying the reference allele was 0.5, and the observed mean proportion was 0.535 using standard alignment methods [14]. The two Drosophila lines differ on average by 3.3 SNPs and 0.74 indels per kb (29,999 SNPs over 5404 genes) [14, 15]. In humans, Panousis et al. (2014) found that 15.6% of variants unequally aligned to the human reference genome [16]. The focal set of European populations in Panousis et al. (2014) had on average 3.53 million SNPs and 546,000 indels (about 1.1 SNPs and 0.17 SNPS per kb) relative to the reference. The studies reviewed so far used a single reference genome. In contrast, Munger et al. 2014 put forth an approach that uses individual genomes as references [17]. They simulated RNA-seq reads from 277 genetically distinct mice, finding that reads which mapped to the individual genome sequences yielded notably different results than reads mapped to a single, reference genome [17]. Out of 6437 true positive cis-acting loci, aligning reads to individual genomes yielded 6349 (98.6%). One percent of all cis-acting regulatory loci (64) were false positives. In contrast, aligning to one reference genome captured 5973 (92.8%) of the true positive cis-acting loci and 1086 false positive loci, 16.9% of the true positive number. The mice lines reported in Munger et al. 2014 had on average 4.6 SNPs per kb and 0.61 indels per kb.

Maize is highly polymorphic, so mapping bias may be common. In an analysis of 916 maize lines, genotypes differed by as many as 60 million variant sites, or about 29 variants per kb [18]. Inbred lines B73 and Mo17 are parents of a high-yielding hybrid and are frequently used in maize genetics research. Chromosomes from Mo17 differ from B73 at about 8 SNPs and 1 indel per kb (7.66 SNPs and 1.11 indels per kilobase) [19]. The first objective of this study was to explore the extent to which estimates of genetic variation for gene expression in maize are affected by preferential read mapping of some genotypes’ RNA-seq reads. Although, there is a large amount of diversity on average, some genomic regions, both large and small, are less variable [20]. Untranslated regions (UTRs) are more variable than coding sequences [21–23]. Splice sites have relatively few SNP differences and are usually highly conserved over very long periods of time [23, 24]. The second objective of this study was to determine if and how preferential read mappings are affected by chromosome and gene level attributes.

We discovered that the effect of mapping bias on maize transcript levels was very strong. Changes in read mapping methodology ameliorated the magnitude of bias, but the number of biased genes was largely unchanged. The degree of preferential mapping differed across splice sites, UTRs, and most notably chromosomal regions. We propose only mapping to individual genomes will ensure the accuracy of maize transcript abundance estimates.

## Results

### Alignment bias affected many genes’ transcript abundance estimates

To evaluate if mapping bias affected maize transcript abundance estimates, we determined if reads from a template genome’s alleles were mapped more often than reads from non-template alleles. We estimated genes’ expression abundances across a population of 105 recombinant inbred maize lines (RILs) derived from a cross between two inbred parents, B73 and Mo17 [25] using STAR and StringTie to align RNA-seq reads and estimate gene coverage [26, 27]. Unlinked loci within this population are in linkage equilibrium. Thus, an association between a gene’s allele and the gene’s expression level across the population, e.g. the presence of a cis-eQTL, indicates allelic variation affects gene expression. 3.47 billion, single-end RNA-seq reads from the 105 lines were aligned to both the B73 and Mo17 genome sequences [19, 28], and gene transcript abundances estimated. If reads from B73 alleles preferentially aligned to the B73 genome relative to reads from Mo17 alleles, RILs with B73 alleles at cis-eQTL would have higher transcript abundances than RILs with Mo17 alleles. Similarly, if reads from Mo17 alleles preferentially aligned to the Mo17 genome, Mo17 alleles would tend to upregulate transcripts relative to B73 alleles. Using the B73 genome as a reference, we detected 9306 cis-eQTL out of 22,408 variable genes. Remarkably, 68.1% (6341) of the eQTL had positive B73 alleles and 31.9% (2965) had positive Mo17 alleles (Table 1). In contrast, using Mo17 the reference genome detected 7985 cis-eQTL from 16,983 variable genes. 64.7% (5169) had positive Mo17 alleles and 35.3% (2816) had positive B73 alleles (Table 1). 50% of the detected eQTL depended on the reference genome. 56% (3525/6341) of the positive B73 cis-eQTL detected when reads were aligned to the B73 reference genome were not detected when Mo17 was used as a reference genome. 43% (2204/5169) of Mo17 positive cis-eQTL detected when reads were aligned to the Mo17 reference genome were not detected when B73 was used as the reference genome.

**Table 1:** Numbers of genes with B73 and Mo17 positively acting cis regulatory alleles using B73 and Mo17 reference genomes revealed read mapping preferences for reference alleles

	B73 reference^a^	Mo17 reference^b^
Total cis-eQTL^c^	9306	7985
B73 positive cis-eQTL	6341 (68.1%)	2816 (35.3%)
Mo17 positive cis-eQTL	2965 (31.9%)	5169 (64.7%)

RNA-seq were mapped to the B73^a^ or Mo17^b^ reference using STAR with the default criteria as given in Supplemental Information.

^c^ The numbers of genes with cis-eQTL were recorded excluding genes with both cis and trans eQTL

**Fig. 1:**
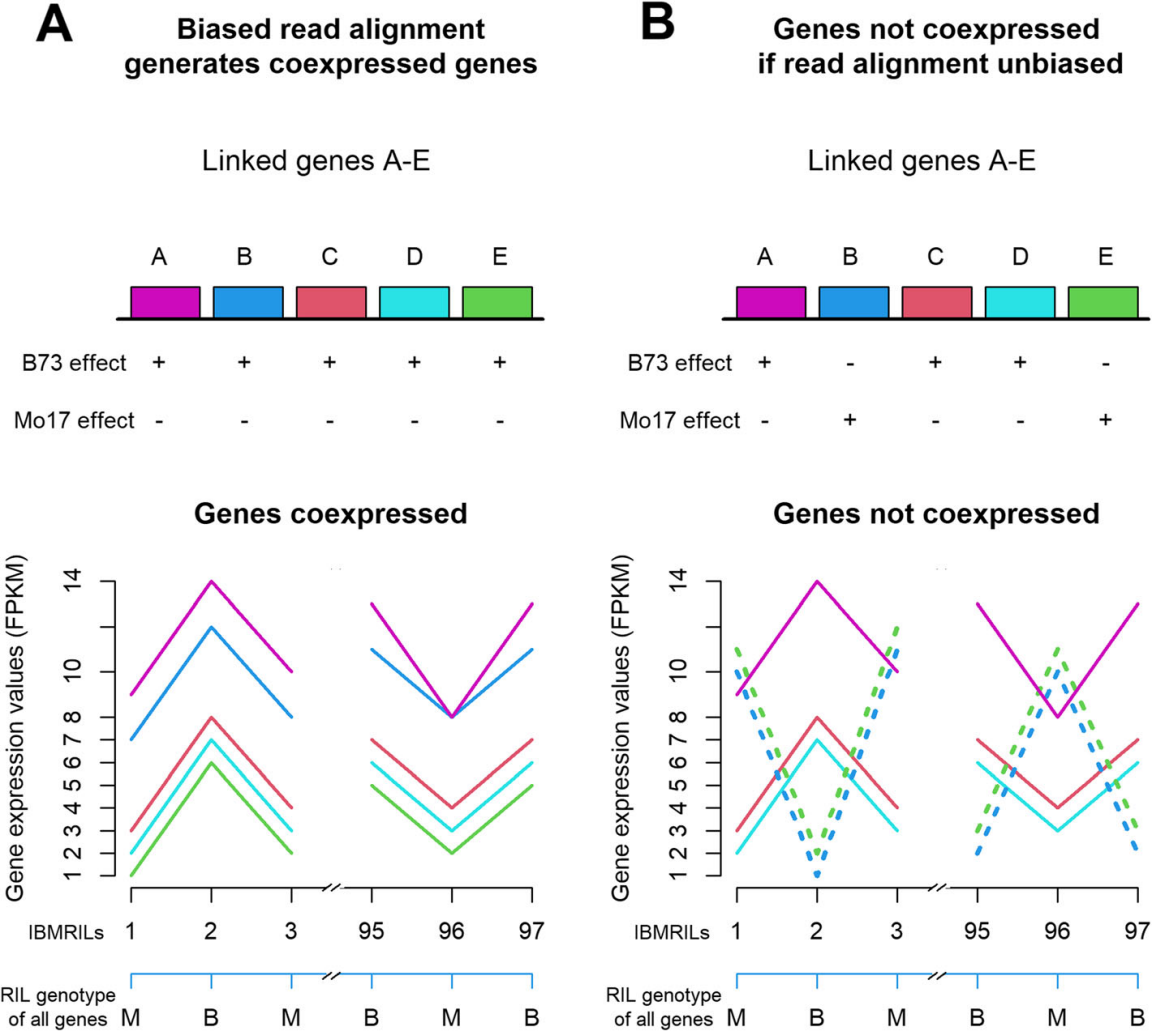
Depiction of a correlation test to determine if preferential alignment affected gene transcript estimates. **a** A chromosomal region whose genes transcripts are all inferred to be correlated across RILs indicates preferential read alignment. A hypothetical region has five linked genes (A-E) that are in complete linkage disequilibrium within the B73 x Mo17 RIL population. Individual members of the RIL population are arbitrarily labelled 1–97. Preferential read alignment causes the B73 allele to have a positive effect on all genes’ transcript abundance estimates relative to the Mo17 allele, leading to one group of coexpressed genes. **b** A chromosomal region with two groups of genes coexpressed across RILs indicates unbiased read alignments across the regions’ genes. Relative to Mo17, B73 alleles increase transcript abundances for genes A, C, and D and decrease transcript abundances for genes B and E. Genes A, C, and D are coexpressed, and genes B and E are also coexpressed

The eQTL analyses identified 1000s of genes whose expression level differences amongst inbred lines depended on the reference genome used to assay expression. Other genes’ expression levels were likely affected but not detected by eQTL mapping because the reference genome had a small effect on transcript level estimates and/or because there was insufficient power to detect a difference between alleles’ transcripts. Since neighboring genes are in strong linkage disequilibrium within the RIL population [29], chromosomal regions are comprised of B73 and Mo17 haplotypes. To detect possible biased alignment amongst genes without eQTL, we determined if genes within haplotypes had correlated transcript levels. In the presence of mapping bias when aligning reads to the B73 reference, linked genes within B73 haplotypes would consistently have higher levels of expression than linked genes within a Mo17 haplotype, and these genes would be coexpressed across the RIL population (Fig. 1a). In contrast, if a B73 haplotype did not have a consistent, positive effect on gene expression, all linked genes would not be coexpressed in a single group (Fig. 1b). The B73 haplotype was inferred to often consistently increase transcript levels of linked genes relative to the Mo17 haplotype. For example, a 132 Mb region of chromosome 10 had one group of 296 correlated genes, the cyan cluster. All genes within this group were positively affected by B73 alleles. At 212 genes, B73 alleles significantly increased transcript expression resulting in cis-eQTL. Similarly, a 20 Mb region of chromosome 3 had one group of 92 coexpressed genes, the plum1 cluster (Tables S1 and S2). All genes within this group were also positively affected by B73 alleles. 79 genes had cis eQTL, and the B73 allele had a positive effect on gene expression for all 79. The large number of genes without eQTL that correlated with linked genes with eQTL (Tables S1 and S2) suggested alignment bias was widespread.

### Sequence divergence between B73 and Mo17 affected transcript abundance estimates

**Fig. 2:**
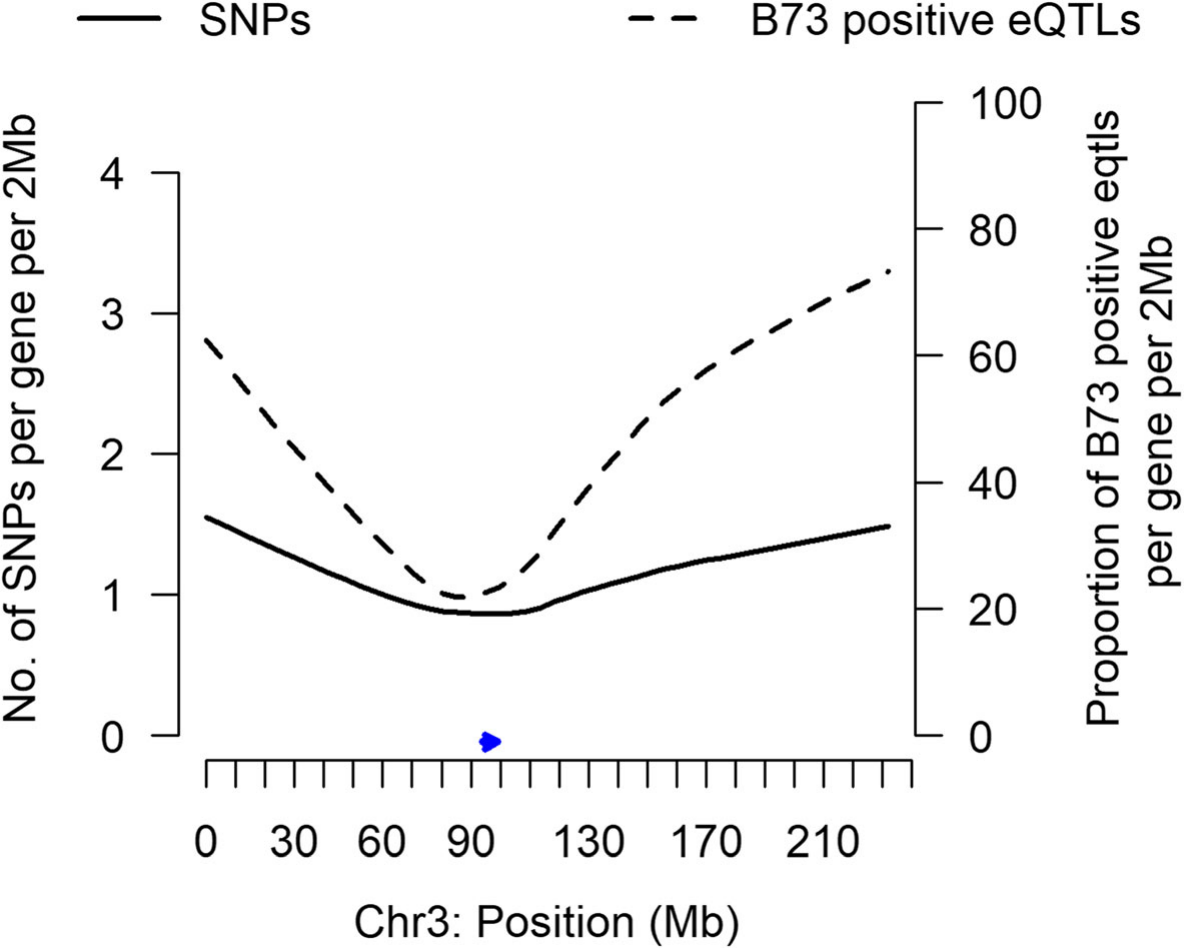
The relationship between the proportion of cis-eQTL that had positive B73 effects when aligned to the B73 reference genome and the number of SNPs between B73 and Mo17 genes. The line plots are a lowess smooth of the number of SNPs per gene per 2 Mb of chromosome 3 sequence and the proportion of B73 positive cis-eQTL per gene per 2 Mb of chromosome 3 sequence. The x-axis is the physical location of intervals in Mb along chromosome 3. The left y-axis is the average number of SNPs within cis-eQTL genes in the intervals. The right y-axis is proportion of B73 positive cis-eQTL out of the total cis-eQTL in the interval. An arrow indicates the centromere location

The frequency of alignment bias greatly increased from chromosomes’ centromeres to telomeres mirroring the occurrence of SNPs between B73 and Mo17. While we could identify SNPs from our RNA-seq data, gene transcript abundance affects SNP calls [30]. Thus, we used SNPs detected by genomic DNA analyses [18]. The occurrence of SNPs between B73 and Mo17 increased from the centromeres to the end of the chromosomes. The correlation between alignment bias and sequence divergence can be seen, for example, in chromosome 3. The proportion of B73 positive eQTL increased from below 40% near the centromeres to close to 80% at one chromosome end (Fig. 2). The number of SNPs per gene increased from an average of 1 SNP around the centromere to an average of 1.5 SNPs at the chromosome ends. Higher alignment bias at divergent chromosome ends can be seen across all chromosomes (Fig. S1).

**Fig. 3:**
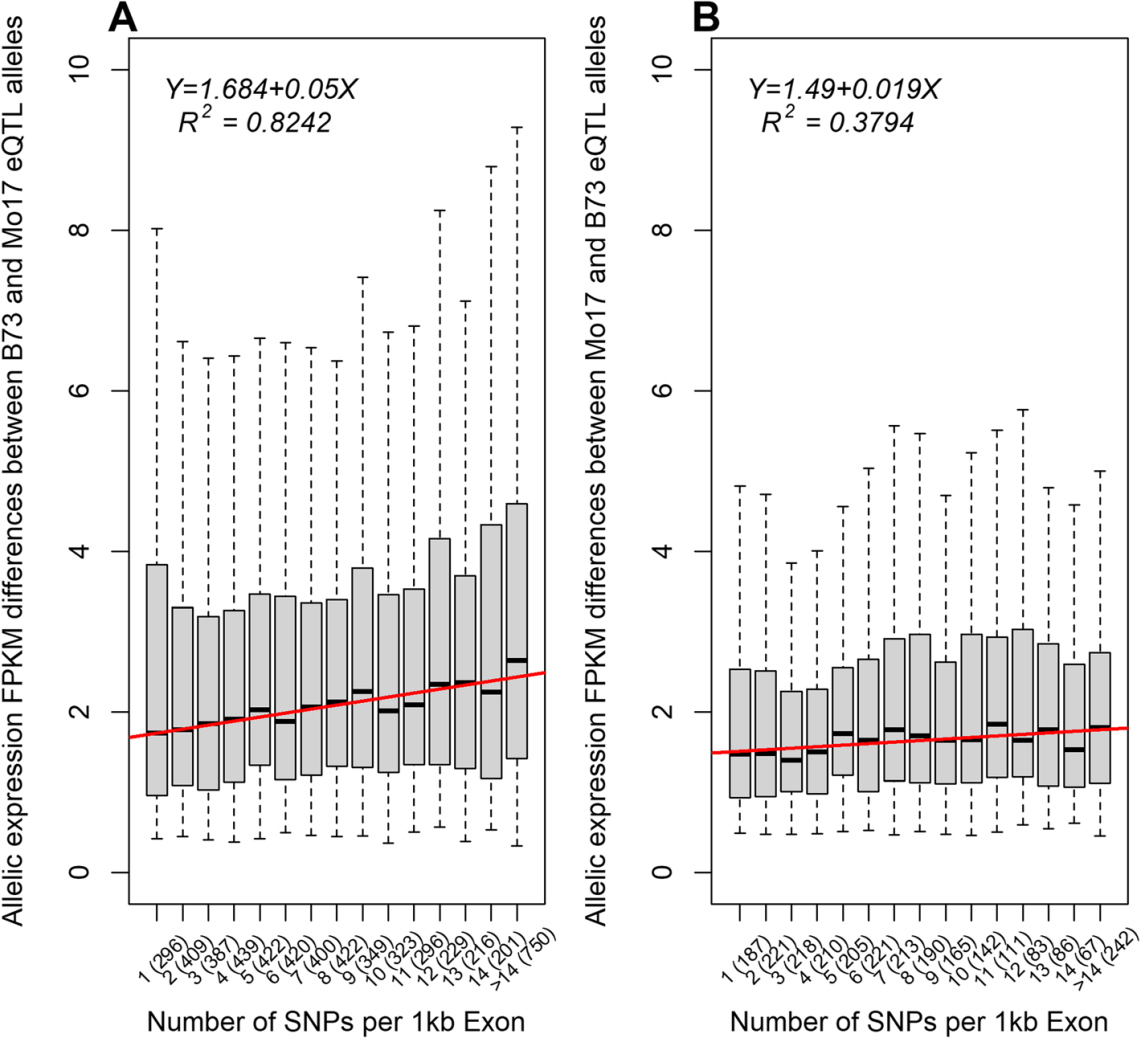
For genes positively affected by B73 (A) and Mo17 (B) cis-eQTL, the number of genic SNPs and expression level differences of homozygous B73 and Mo17 lines were plotted. Exon sequence divergence was calculated as the number of SNPs matching exons in a gene divided by the total exon lengths and multiplied by 1000. The numbers in parenthesis represent the numbers of genes in each group. The y-axis is the absolute value of the allelic expression level difference of B73 minus Mo17 in FPKM, or two times the expected additive effect of the locus. **a** Expression differences between B73 and Mo17 were highly correlated with numbers of SNPs per 1 kb exon for the 5559 B73 positive cis-eQTL genes with SNP information. **b** Expression level differences between Mo17 and B73 were weakly correlated with numbers of SNPs per 1 kb exon for the 2561 Mo17 positive cis-eQTL genes

Nucleotide sequence divergence positively correlated with transcript level differences between the B73 reference alleles and the Mo17 non-reference alleles. We correlated B73 and Mo17 SNPs detected by genomic DNA analyses with allelic differences in gene expression [18]. Of the 6341 genes upregulated by B73 cis-eQTL, 5559 had one or more SNP. Median fragments per kilobase exon per million mapped reads (FPKM) difference between individuals homozygous for alternative alleles increased by 0.05 FPKM per SNP per 1 kb exon (Fig. 3a, *P* = 2.9e-6, *R*^*2*^ = 0.82). Transcript abundances in divergent sequences may differ because sequence divergence can correlate with regulatory divergence [31]. To investigate this idea, we examined the effects of sequence divergence on Mo17 reads aligned to the B73 reference genome. Among Mo17 positive eQTL, the expression differences between Mo17 genes dissimilar to B73 genes were only slightly higher than differences between the Mo17 genes similar to B73 genes (Fig. 3b, *P* = 0.014, *R*^*2*^ = 0.38). Preferential mapping of B73 reads to the B73 genome at diverged loci best explained the positive relationship between sequence divergence and expression divergence amongst eQTL.

Preferential mapping of reads was also higher across UTRs relative to other gene features. Among the genes with positive B73 cis-eQTL effects, a median of 15.7% of reads mapped to UTRs. In contrast, among the genes with negative B73 cis-eQTL effects, the median was 13.4% (Wilcoxon rank sum test, W = 25,439,952, *P* < 2.2e-16; Table 2). Furthermore, among those genes that had B73 alleles that enhanced expression, reads from B73 alleles aligned more to UTRs than did reads from Mo17 alleles. Among these genes, 17.1% of genes’ reads from B73 mapped to UTRs. By contrast, 14.3% of reads from Mo17 mapped to UTRs (Wilcoxon signed rank test, V = 7,544,600, *P* < 2.2e-16, Table 2).

**Table 2:** Median percentages of reads from B73 and Mo17 that mapped to UTRs relative to exons among genes upregulated by B73 or Mo17 eQTL

	B73 reads	Mo17 reads	All reads^c^
B73 + eQTL	17.1^a^	14.3	15.7
Mo17 + eQTL	13.7^b^	13.1	13.4

^a^Among genes upregulated by B73 alleles at cis eQTL, the percentage of reads that mapped to UTRs was significantly higher for reads derived from B73 alleles than for reads derived from Mo17 alleles. Wilcoxon signed rank test, V = 7,544,600, *P* < 2.2e-16

^b^Among genes upregulated by Mo17 alleles at cis eQTL, the proportion of reads that mapped to UTRs was also significantly higher for reads derived from B73 alleles than for reads derived from Mo17 alleles. Wilcoxon signed rank test, V = 1,324,300, *P* = 1.1e-8

^c^The percentage of all reads irregardless of allelic origin that mapped to UTRs was significantly higher for genes upregulated by B73 alleles than genes with upregulated by Mo17 alleles. Wilcoxon rank sum test, W = 25,439,952, *P <* 2.2e-16

Genes with biased read alignments had a low proportion of reads aligning to splice junctions. Transcript levels of genes with more junction spanning reads were less affected by preferential alignment than genes with fewer. Genes with B73 positive eQTL had a very small but significantly lower percentage of reads that spanned splice junctions than did reads with Mo17 positive eQTL (15.2% vs. 15.7%; Wilcoxon rank sum test, W = 23,696,329, *p*-value = 4.518e-4; Table 3). In addition, across the genes with splice sites for which Mo17 alleles enhanced expression, reads from Mo17 alleles overlapped splice sites at a small yet significantly higher percent than reads from B73 alleles (15.8 vs. 15.6; Wilcoxon signed rank test, V = 1,269,200, *p*-value = 0.019; Table 3).

**Table 3:** Median percentages of reads from B73 and Mo17 that mapped to splice junctions relative to all exons for genes with B73 or Mo17 positive eQTL

	B73 reads	Mo17 reads	All reads^c^
B73 + eQTL	15.2^a^	15.3	15.2
Mo17 + eQTL	15.6^b^	15.8	15.7

^a^Among genes upregulated by B73 alleles, the proportion of reads that mapped to splice sites was significantly higher for Mo17 derived reads than B73 derived reads. Wilcoxon signed rank test, V = 5,474,100, *p-*value = 4.947e-12

^b^Among genes upregulated by Mo17 alleles, the proportion of reads that mapped to splice sites was significantly higher for Mo17 derived reads than B73 derived reads. Wilcoxon signed rank test, V = 1,269,200, *p*-value = 0.019

^c^The percentage of all reads that mapped to splice sites was significantly lower in genes upregulated by B73 alleles compared to genes upregulated by Mo17 alleles. Wilcoxon rank sum test, W = 23,696,329, *p-*value = 4.518e-4

### Read alignment parameters and software reduced the effects of preferential read alignments, but all analyses detected a large number of biased genes

**Fig. 4:**
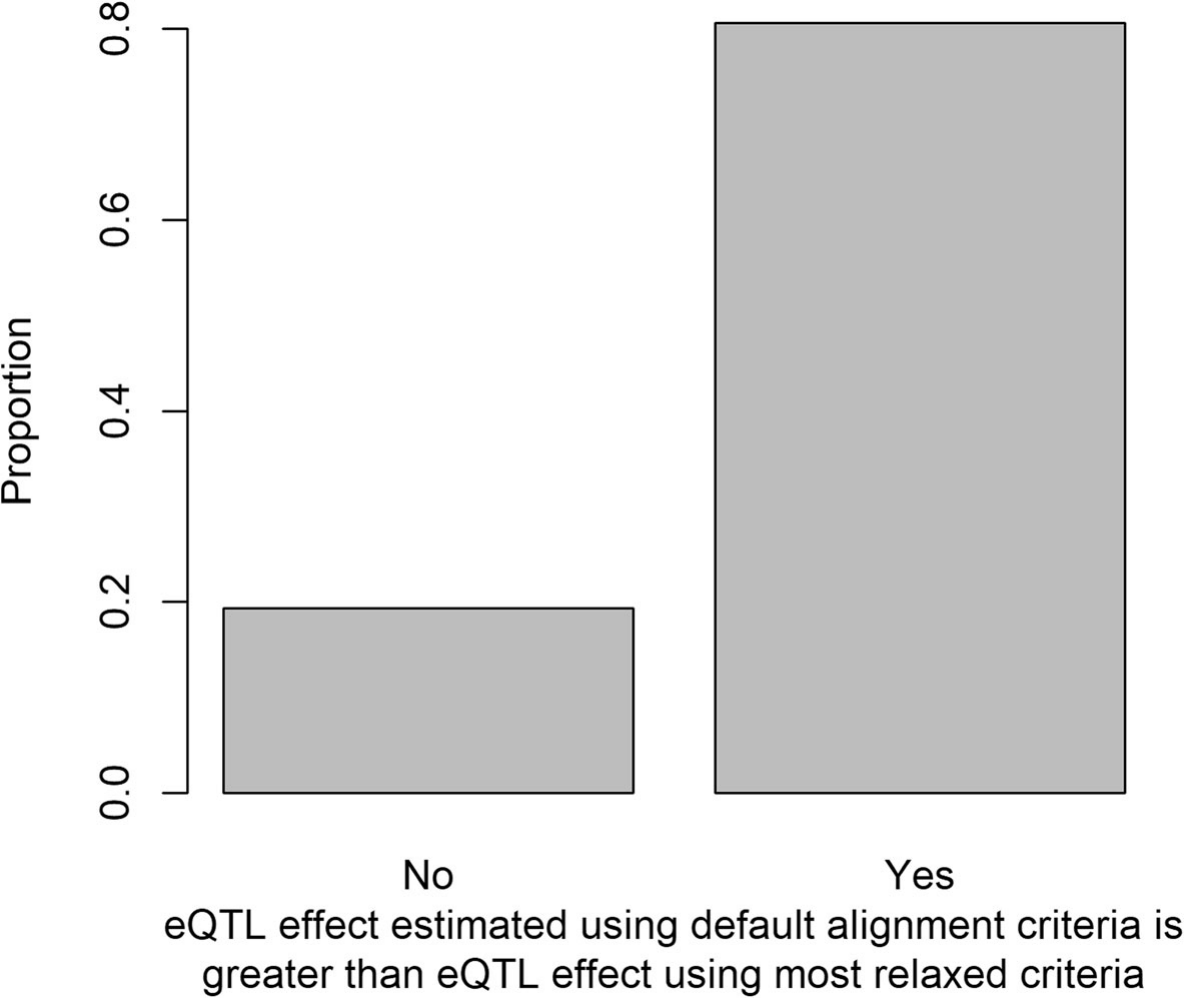
Comparison of alignment criteria on cis-eQTL effect size. Each cis-eQTL for which the B73 allele increased a target gene’s expression was labelled “yes” if its effect estimated using the most relaxed alignment criteria was lower than its effect using the default alignment criteria. The proportion of “yes” genes was calculated. The cis-eQTL effect was estimated as the average of the mean expression of RILs with the B73 genotype minus the mean expression of RILs with the Mo17 genotype. A large proportion of B73 positive cis-eQTLs had greater effects using default alignment criteria (binomial test, *P* < 2.2e-16)

Allowing more SNPs between reads and the genome template reduced the effects of preferential read alignment. New eQTL identified when allowing more SNPs in alignment criteria had significantly more Mo17 positive effects than B73 positive effects relative to existing eQTL identified with default criteria (Table S3, X^2^-test, *P* < 2.2e-16). The effects of eQTL also changed. 5951 eQTL at which B73 alleles promoted gene expression were shared between analyses that used gene expression estimates calculated by the default and most relaxed mapping criteria. 4801 (80.7%) had greater effects on transcript abundance in the default analysis (Fig. 4). Similarly, among the 6005 cis-eQTL detected in analyses of both the default and more relaxed alignment datasets, 4872 (81.1%) had greater B73 effects in the default analysis (Fig. S2).

Changing alignment criteria had little effect on the numbers of genes with biased read alignment. B73 alleles promoted expression at 66.6% of eQTL detected with the most relaxed alignment criteria. This proportion was not significantly lower than the 68.1% found using default criteria (X^2^-test, *P* = 0.03, Table 4).

**Table 4:** Number of genes with B73 and Mo17 positively acting, cis-regulatory alleles detected in different analyses

Methods	STAR-StringTie			TopHat2-Cufflinks			Salmon
cis-eQTL only	default criteria	relaxed criteria	most relaxed criteria	default criteria	relaxed criteria	most relaxed criteria	default criteria
Total	9306	9256	9260	11,908	11,720	11,643	7855
B73 positive cis-eQTL	6341 (68.1%)	6203 (67.0%)	6172 (66.6%)	9905 (83.2%)	9639 (82.1%)	9504 (81.6%)	4959 (63.1%)
Mo17 positive cis-eQTL	2965 (31.9%)	3053 (33.0%)	3088 (33.3%)	2003 (16.8%)	2081 (17.8%)	2139 (18.4%)	2896 (36.9%)

Many studies have reported genetic variation for gene expression using TopHat and Cufflinks analyses [32]. Although the STAR read alignment and StringTie transcript assembly analyses detected large numbers of genes whose expression values were due to preferential read mapping, TopHat and Cufflinks transcript estimates were even more affected. Among the 28,289 genes with the most variable expression, 11,908 were significantly affected by one cis-eQTL and no trans-eQTL (Table 4). For 83.2% (9905) of these genes, the B73 cis-eQTL upregulated transcript levels (Table 4). Among detected cis-eQTL, sequence divergence explained almost all variation in median allele expression differences (Fig. S3, *P* = 4.4e-10, *R*^*2*^ = 0.95). Alignment bias affected genes in addition to those with cis eQTL. Genes in many chromosomal regions were solely upregulated by B73 alleles and thus correlated across the RIL population (Fig. 1, Tables S4 and S5). As with the STAR/StringTie analyses, allowing more SNPs in alignments enabled more reads to align, but preferential alignment remained widespread. 87.0% (7826 of 8999) of the B73 positive cis-eQTL detected from analyses of gene expression from both the default and the most relaxed alignment criteria had higher effects in the former. Nonetheless, the number of genes affected by bias was largely unchanged. Reducing stringency reduced the total number of genes with cis eQTL by only 2% (265 genes) and the proportion of B73 positive eQTL by only 1.6% (Table 4).

Analyses of gene expression estimates calculated with salmon, an alignment free based approach to map reads to transcripts [33], also revealed preferential read mapping. Salmon estimated gene transcript abundances from reads mapped to B73 transcripts. Among the genes that varied across the RIL population, B73 alleles upregulated 63.1% (4959/7855) (Table 4).

## Discussion

### Large-scale alignment bias in maize may indicate frequent misestimation of allelic effects on gene expression

Our results show that in maize mapping bias had a strong effect on transcript abundance estimates and was a major, genome-wide driver of eQTL detection. About 50% of cis-eQTL that were detected depended on the reference genome template that was used (Table 1), and many genes not associated with cis-eQTL likely had preferential alignment of one parent’s alleles (Fig. 1; Tables S1 and S2). Previous investigations of genetic variation for maize gene expression may have included high proportions of false positives. For example, we utilized RNA-seq data from Li et al. 2013. Li et al. estimated gene transcript abundance by aligning reads to the B73 genome using a method very similar to our default method, so 50% of eQTL reported in Li et al. were potentially false positives. Similarly, Wang et al. (2018) aligned reads from maize and its wild progenitor teosinte to the B73 genome. Wang et al. reported maize eQTL alleles condition higher expression than do teosinte alleles [34] and reported linked genes had cis-eQTL with the same, positive acting allele [34]. While Wang et al. interpreted these results as biologically relevant, they may at least partially be due to alignment bias both genome-wide (Table 1) and within chromosomal domains (Fig. 2, Table 2, Fig. S1).

The very large effects of alignment bias on transcript estimates would likely occur in other species that are highly variable. In *Arabidopsis thaliana*, accession Can-0 harbors 6.63 SNPs/kb and 1.76 indels/kb compared to the reference accession Col-0 [35]. A wild-derived mouse strain MOLF/EiJ contains 6.34 SNPs/kb and 0.96 indels/kb relative to the reference strain C57BL/6 J [36]. Two different specimens of the nematode *Caenorhabditis brenneri* differ on nearly every sixth base pair on average [37].

Remarkably, preferential mapping in maize was unevenly distributed across chromosomes and scaled with chromosomal features’ sequence divergence. Highly divergent chromosome arms had much more frequent mapping biases than more similar pericentromeric chromosomal regions (Fig. 2; Fig. S1). This chromosomal effect on mapping bias may occur in other cereals including rice and wheat that have large pericentromeric regions with low crossover frequencies and low diversity relative to terminal regions [38, 39]. In plant breeding, large chromosomal segments from an unrelated species also may be introgressed into elite germplasm [40].

Reads that aligned to splice site junctions, whose sequences evolve slowly, were slightly but significantly underrepresented amongst genes with biased alignments (Table 3). Genes’ UTRs are more divergent than other genic regions, and reads that align to UTRs were also slightly but significantly enriched amongst genes with preferential read alignment (Table 2). While differences in UTR and splice site read alignments were small between B73 and Mo17 alleles, other, relatively short genome sequence domains likely have greater effects on gene expression across genotypes. For example, NB-LRR genes and other biotic response genes are highly polymorphic compared to housekeeping genes [41].

### The high level of maize alignment bias may be due to structural variants that can be accounted for by de novo genome assemblies

Increasing the number SNPs allowed in alignments to the B73 genome by relaxing criteria caused the effect sizes of 4/5 B73 positive eQTL to decrease (Fig. 4). Nonetheless allowing more SNPs within alignments did little to resolve biased alignment. The total number of B73 positive eQTL was similar regardless of alignment criteria changes (Tables 1 and 4). Structural variants including translocations, inversions, indels, and copy number variants, likely contributed most to biased alignment. Between B73 and Mo17, structural variants occur within about ~ 20% of maize genes, and 10% of annotated genes are exclusive to one or the other inbred line [19]. In humans, eQTL were enriched 50-fold amongst genes with structural variants relative to genes with SNPs or short indels [42]. Structural variants would cause reads not to align to a reference genome or to misalign to the reference. While in mice reads that did not map to a reference occurred about 20 times more often than reads that mismapped to the reference [17], maize has a large number of closely related gene and pseudogene sequences because of genome duplication and transposon activity [19, 28]. Non-target reference genome sequences may improperly capture a relatively high proportion of reads from non-reference genotypes [43].

Software notably affected read alignment bias magnitudes (Tables 1 and 4). For example, while 68.1% of eQTL had B73 alleles that increased expression in STAR/StringTie analyses, 83.2% did in TopHat/Cufflinks analyses, and 63.1% did in Salmon analyses. STAR’s alignment algorithm accurately assigns a higher proportion of reads to a template than does TopHat2’s [44], and StringTie provides better estimates of gene expression abundances compared to Cufflinks [27]. Salmon’s accounting for factors that affect read coverage such as fragment GC content and 5′ and 3′ sequence bias may have increased the similarity of different alleles’ expression values relative to STAR/ StringTie [33]. Steps such as removing UTRs from a reference template prior to alignment (Table 2), masking polymorphic sites [34], and extending reads [45] would generate more accurate transcript abundance estimates. Cho et al. 2014 showed that compared to short paired reads, long paired reads have a higher unique alignment rate; have a higher chance of differentiating haplotypes at heterozygous sites; and more accurately quantified transcript abundances [45]. Thus, we anticipate modified genome templates, novel software, and longer sequencing reads will reduce biased transcript level estimates.

Nonetheless, these methods are partial solutions in the presence of structural variants [46]. De novo genome sequencing is becoming more routine, comprehensive, and accurate because of long-read sequencing, long paired-end reads, and optical mapping [47–51]. Individual genomes can be inferred from a small number of core genome sequences [17], reducing sequencing and bioinformatics burdens. These distinct genome references will enable reads from sequences affected by structural rearrangements to correctly align enabling accurate estimates of gene expression across genotypes.

## Conclusions

In conclusion, read mapping bias greatly affects transcript abundance estimates in maize genotypes. Transcript level misestimation is much greater for genes at chromosome ends than in pericentromeric regions, and its misestimation is slightly but significantly higher for genes with many UTR sourced reads. Alignment parameters and software reduced the magnitude of mapping bias but had little effect on its widespread presence. Previous studies of genetic variation of gene expression for maize and other species with highly divergent chromosomal sequences were likely strongly affected by read mapping bias. Assigning reads to individual genomes or transcriptomes is likely necessary to consistently and accurately estimate genotypes’ transcript abundances.

## Methods

### Gene expression estimation, genetic map construction, and eQTL mapping

A recombinant inbred line population derived from selfing F_2_ plants from a biparental cross is an excellent resource for identifying alleles that affect gene expression abundance. Each allele is expected to be within 50% of the population, so allelic comparisons are well-powered. In addition, unlinked loci are in linkage equilibrium, so an association between an allele and a gene’s abundance signifies the region causes the expression variation. The RNA-seq data was composed of raw 103-110 nt single end RNA-seq reads without barcodes or adapters from 105 maize B73 x Mo17 RILs and two parents. Data were downloaded from NCBI accession SRA055066 [25]. Details on plant growth, RNA extraction, and sequence generation are given in Li et al. 2013. We trimmed low quality 3′ sequences using fastx_trimmer (http://hannonlab.cshl.edu/fastx_toolkit/).

Genes’ transcript levels were estimated for the parental lines and 97 RILs eight times. Eight of the 105 RILs were excluded because of high heterozygosity and missing marker calls. The first analysis was with the Mo17 genome Zm-Mo17-REFERENCE-CAU-1.0 [19]. STAR-2.5.3a aligned reads using default parameters as listed in Table S6; StringTie-1.3.3b estimated gene transcript abundances. The next six analyses used alignments to the B73 reference genome AGPv3.19 [28]. In the first three analyses, STAR-2.5.3a software was used with three parameter sets, default, relaxed, and most relaxed, which allowed 3, 6 and 12 nucleotide mismatches to the template, respectively (Table S6), and StringTie estimated transcript abundances. TopHat-2.1.1 and Cufflinks-2.1.1 software estimated transcript abundances in the second three analyses with parameter sets analogous to those used in the STAR alignment (Table S6). In these first seven analyses, samtools-1.3.1 *rmdup* removed duplicate reads, and gene transcript abundance was reported as FPKM. Analysis of TPM estimates generated by StringTie with STAR using the default parameters revealed an almost identical degree of read mapping bias as did FPKM analysis (Table S7). Salmon 1.3.0 was used for the eighth analysis. Trimmed reads were mapped to the AGPv3.19 transcriptome, and transcript abundances were translated to gene level expression abundances using Tximport-1.18.0.

To construct a genetic map for eQTL mapping, we used samtools-1.3.1 mpileup to collect nucleotide information from the bam files produced by aligning B73, Mo17 and RIL reads to the B73 reference with Tophat2 default criteria. BCFtools-1.3.1 called variants. We identified 25,822 SNPs between B73 and Mo17, requiring nucleotide calls to have > = 5 reads with phred-scaled quality scores of > = 20. We used these SNPs to genotype RILs. To be included in the genetic map, SNP markers had to have a minor allele frequency > 30% and a missing data rate < 5%. MadMapper (https://github.com/alex-kozik/atgc-map) and MSTmap [52] grouped and ordered markers, respectively, producing a map with 2236 markers separated by 0.1 to 31.5 cM. The total map length was 3606 cM. Markers were distributed throughout the linkage groups corresponding to the 10 maize chromosomes without any breaks, except chromosome 7 was represented by two linkage groups. As expected, the SNP genotyping revealed that individuals have chromosomes composed of long stretches of B73 and Mo17 DNA. There were no cases where a Mo17 allele was detected in a B73 region or visa- versa.

Using this genetic map, eQTL mapping was conducted with each of the 8 different gene expression datasets. We first selected genes expressed at > 1 FPKM or > 1.82 TPM in at least 42 RILs (43%) and subsequently selected the 75% most variable genes among the RILs. eQTL were mapped using non-parametric interval mapping in R/qtl [53] (version 1.36–6). 1000 permutations were used to define a genome-wide LOD significance threshold for an eQTL at α = 0.05. eQTL linked to the target gene were deemed cis-eQTL [54], and all other eQTLs were termed trans-eQTL. The average gene expression of lines carrying the B73 and Mo17 cis-eQTL alleles were estimated using the *effectplot* function in R/qtl. Cis-eQTL magnitude was expressed as half of the difference between the two alleles.

#### Correlating sequence divergence of eQTL genes with eQTL magnitudes

To determine the relationship between sequence divergence and expression divergence, we downloaded the *Zea mays* haplotype map, HapMap v3.2.1 [18] (https://www.panzea.org/genotypes), and extracted the B73 and Mo17 SNPs. We mapped the SNPs to exons annotated in the B73 reference genome AGPv3.19, selected those exons to which RNA-seq reads mapped, and calculated the number of SNPs per 1 kb exon. We used SNPs from the haplotype map instead of the RNA-seq data to have a complete set of independently measured SNPs. To map SNP frequencies across chromosomal regions and compare them to eQTL (Fig. 2), we captured consecutive, non-overlapping windows of 2 Mb. Within the window, we averaged the numbers of SNPs per gene and calculated the proportion of genes with eQTL that had positively acting B73 alleles. We plotted results from the loess.smooth() function in R for each chromosome. To evaluate the significance of the relationship between SNPs and eQTL magnitudes (Fig. 3), we modeled median eQTL magnitude as a linear function of SNP number using R.

#### Identification of co-expressed linked genes across the RIL population

One expects mapping bias would affect genes in addition to those detected in eQTL analyses. We investigated if a region’s haplotype had a consistent effect on the region’s gene expression values (Fig. 1). To determine if a chromosomal region’s genes were positively correlated, we used the R package WGCNA-1.49 [55, 56], with a soft threshold power, β, of 5 to identify correlated gene groups. The STAR/StringTie and TopHat2/Cufflinks default criteria expression datasets used for the eQTL analyses were used for coexpression analyses. Default WGCNA settings were used to define groups of genes, except groups with fewer than 60 genes and/or higher than 0.8 topological overlap (TO) similarity were merged into the closest neighbour group. We determined the chromosomal positions of genes within groups, and we identified the haplotype allele that increased the expression levels of group’s genes.

#### Determining if reads’ UTR and splice site usage affected eQTL bias

We used similar approaches to test if splice sites and UTRs affected preferential read alignment. In both cases, we used processed RNA-seq reads of the B73 and Mo17 inbred lines aligned to the B73 genome with STAR-2.5.3a with default alignment criteria. For each gene with a cis-eQTL, the SGSeq R package recorded the number of B73 and Mo17 reads overlapping annotated splice junctions and all exons [57]. For each line, we calculated each gene’s splice junction read ratio, the ratio of splice junction mapped reads relative to the total mapped reads. Only genes with reads mapping across splice junctions and other genic regions were considered. The Bioconductor package Rsubread was used to count RNA-seq reads mapping to exons and UTRs. For each cis-eQTL gene, we calculated the ratio between the number of reads mapping to UTRs and the number of reads mapping to all exons for B73 derived reads and Mo17 derived reads [58]. Only genes with reads mapping to UTRs and CDS sequences were evaluated. The median difference in splice junction read ratio and the median difference in UTR read ratio between the B73 inbred and the Mo17 inbred were calculated and the significance evaluated using the Wilcoxon signed rank test for genes with cis-eQTL. A similar analysis was done on the subset of eQTL genes for which the B73 cis-eQTL allele was positive and the subset for which the Mo17 cis-eQTL allele was positive. The significances of the median differences in splice junction and UTR read ratios between B73 cis-eQTL genes and Mo17 cis-eQTL genes were calculated using the Wilcoxon rank sum test.

#### Code availability

Code used in the study is available from GitHub: https://github.com/lewislukens/maizeeqtlstudy and in a supplementary file: codeProtocols.txt (Additional file 11).

## Supplementary Information

**Additional file 1: Fig. S1** The relationship between the proportion of cis-eQTL for which B73 alleles had positive effects on gene expression when aligned to the B73 reference genome and the number of SNPs between B73 and Mo17 genes. The line plots are lowess smooths of the number of SNPs per gene per 2 Mb of a chromosome sequence and the proportion of B73 positive cis-eQTL per gene in 2 Mb of chromosome sequence. The x-axis is the physical location in Mb along a chromosome. The left y-axis is the average number of SNPs within cis-eQTL genes in 2 Mb intervals. The right y-axis is the proportion of B73 positive cis-eQTL out of all cis-eQTL in a 2 Mb interval. Arrows indicate the centromere locations.

**Additional file 2: Fig. S2** Comparison of alignment criteria on cis-eQTL effect size. Each locus for which the B73 increased a target genes expression was labelled “yes” if the B73 effect estimated using the more relaxed alignment criteria was lower than its effect using the default alignment criteria. The proportion of “yes” genes was calculated. Cis-eQTL effect is estimated as the average of the mean expression of genotype B73 minus the mean expression of genotype Mo17. A large proportion of B73 positive cis-eQTLs have greater effects using default alignment criteria (binomial test, *P* < 2.2e-16).

**Additional file 3: Fig. S3** For genes with B73 positive cis-eQTL when aligned to the B73 genome by TopHat2 with the default parameters, the expression level differences of homozygous B73 and Mo17 lines were plotted relative to SNP frequency. Exon sequence divergence was calculated the number of SNPs matching exons in a gene divided by the total exon lengths and multiplied by 1000. The numbers in parenthesis represent the numbers of genes in each group. Allelic expression differences between B73 and Mo17 were highly correlated with numbers of SNPs per 1 kb exon for the 8794 B73 positive cis-eQTL genes with SNP information. The y-axis is the allelic expression level difference of B73 minus Mo17 in FPKM, or two times the expected additive effect of the locus.

**Additional file 4: Table S1** The chromosomal locations of and parental allele contributions to genes coexpressed across RILs.

**Additional file 5: Table S2** Genetic and physical map locations of physically and genetically linked coexpressed genes.

**Additional file 6: Table S3** A comparison of the numbers of cis-eQTL genes detected with the default alignment data and cis-eQTL genes detected with relaxed and most relaxed alignment data.

**Additional file 7: Table S4** The chromosomal locations and parental allele contributions to genes coexpressed across RILs from TopHat2-Cufflinks analyses with the default parameters.

**Additional file 8: Table S5** Genetic and physical map locations of physically and genetically linked coexpressed genes from a TopHat2-Cufflinks analysis with the default parameters.

**Additional file 9: Table S6** Different sets of varying alignment parameters used for TopHat2 and STAR against maize reference genomes.

**Additional file 10: Table S7** Numbers of genes with B73 and Mo17 positively acting cis regulatory alleles using gene expression TPM and FPKM metrics from STAR-StringTie analyses with the default parameters revealed read mapping preferences for reference alleles.

**Additional file 11: codeProtocols.txt.**

## Data Availability

All the necessary information needed to support the results of this paper are included within the article. All of the RNA sequencing data used in the study were deposited to the NCBI SRA database (https://www.ncbi.nlm.nih.gov/sra) under the accession number SRA055066 by [23].

## Abbreviations

RNA-seq: RNA sequencing
SNP: Single Nucleotide Polymorphism
Indels: Insertions/deletions
cis-eQTL: Cis-acting expression quantitative trait loci
trans-eQTL: Trans-acting expression quantitative trait loci
UTRs: Untranslated regions
FPKM: Fragments per kilobase of exon model per million reads mapped
TPM: Transcripts per kilobase million
NB-LRR: Nucleotide-binding and leucine-rich repeat
SVs: Structural variants
RILs: Recombinant inbred lines
SRA: Sequence Read Archive
