# Supplementary figures and images for: Zea mays RNA-seq estimated transcript abundances are strongly affected by read mapping bias
Source: BMC Genomics. 2021 Apr 20;22:285. doi: 10.1186/s12864-021-07577-3 (PMC8056621; doi:10.1186/s12864-021-07577-3)

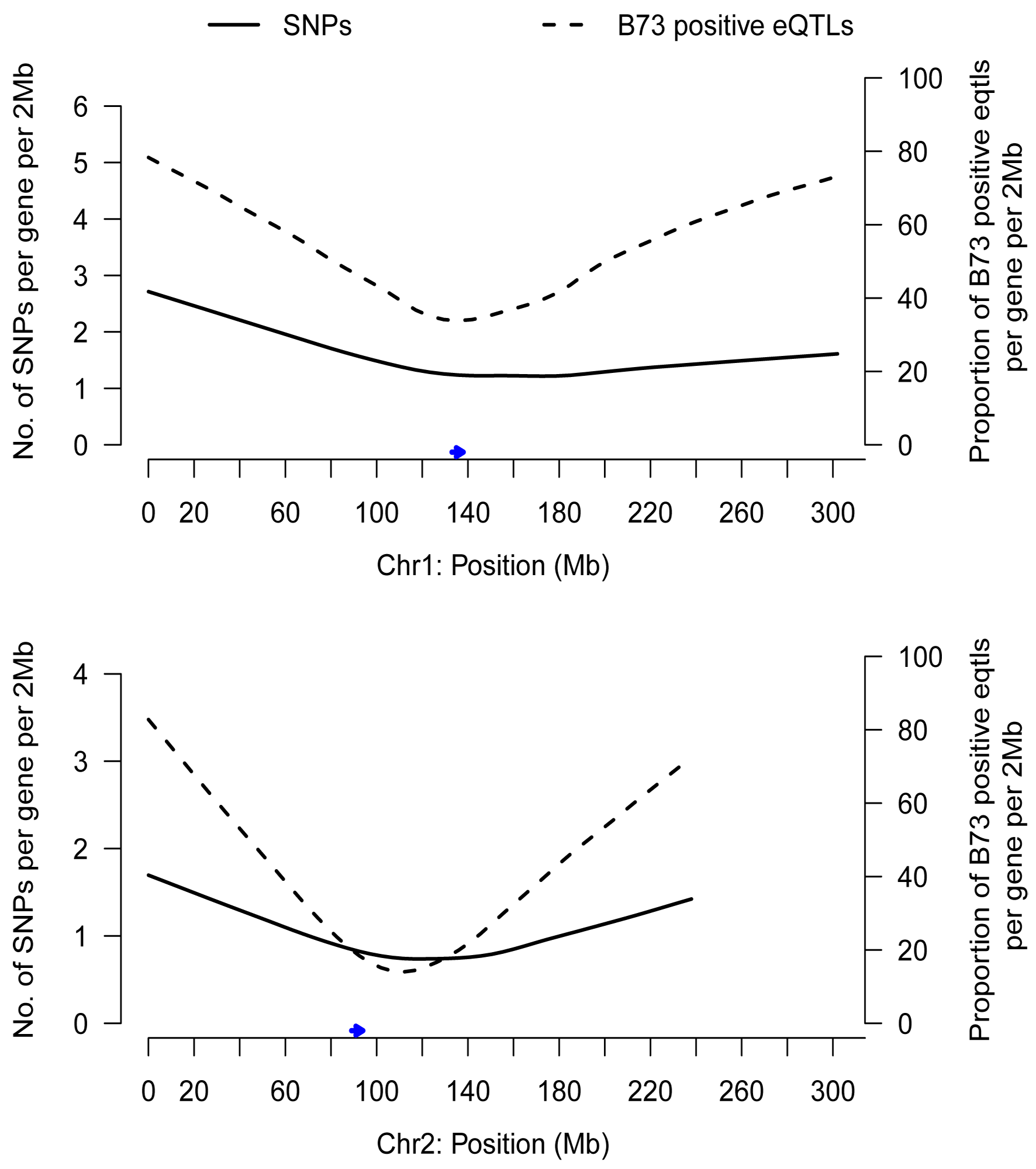

Supplement: Supplementary file 1 — Additional file 1: Fig. S1 The relationship between the proportion of cis-eQTL for which B73 alleles had positive effects on gene expression when aligned to the B73 reference genome and the number of SNPs between B73 and Mo17 genes. The line plots are lowess smooths of the number of SNPs per gene per 2 Mb of a chromosome sequence and the proportion of B73 positive cis-eQTL per gene in 2 Mb of chromosome sequence. The x-axis is the physical location in Mb along a chromosome. The left y-axis is the average number of SNPs within cis-eQTL genes in 2 Mb intervals. The right y-axis is the proportion of B73 positive cis-eQTL out of all cis-eQTL in a 2 Mb interval. Arrows indicate the centromere locations. [file 12864_2021_7577_MOESM1_ESM.tif]

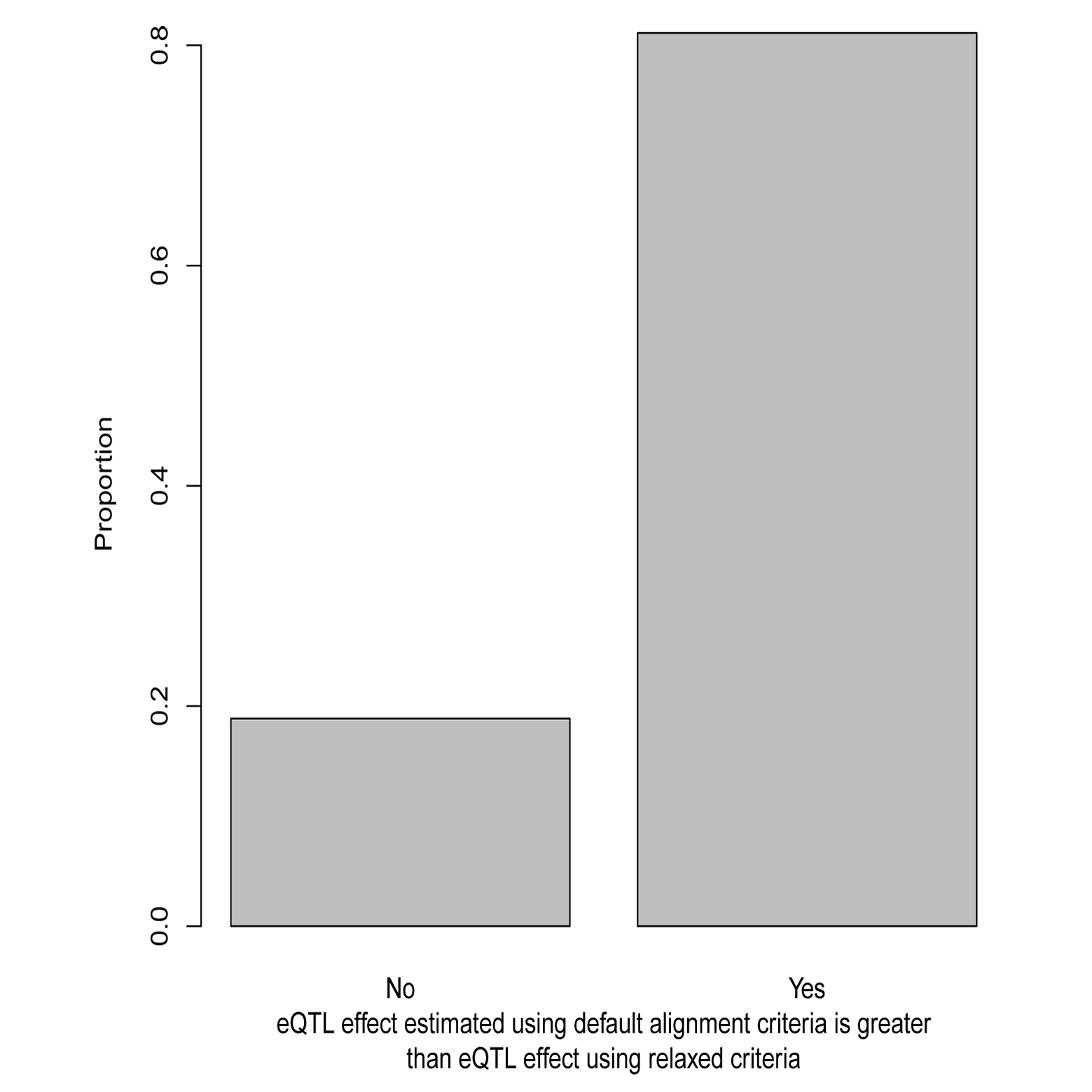

Supplement: Supplementary file 2 — Additional file 2: Fig. S2 Comparison of alignment criteria on cis-eQTL effect size. Each locus for which the B73 increased a target genes expression was labelled “yes” if the B73 effect estimated using the more relaxed alignment criteria was lower than its effect using the default alignment criteria. The proportion of “yes” genes was calculated. Cis-eQTL effect is estimated as the average of the mean expression of genotype B73 minus the mean expression of genotype Mo17. A large proportion of B73 positive cis-eQTLs have greater effects using default alignment criteria (binomial test, P < 2.2e-16). [file 12864_2021_7577_MOESM2_ESM.tif]

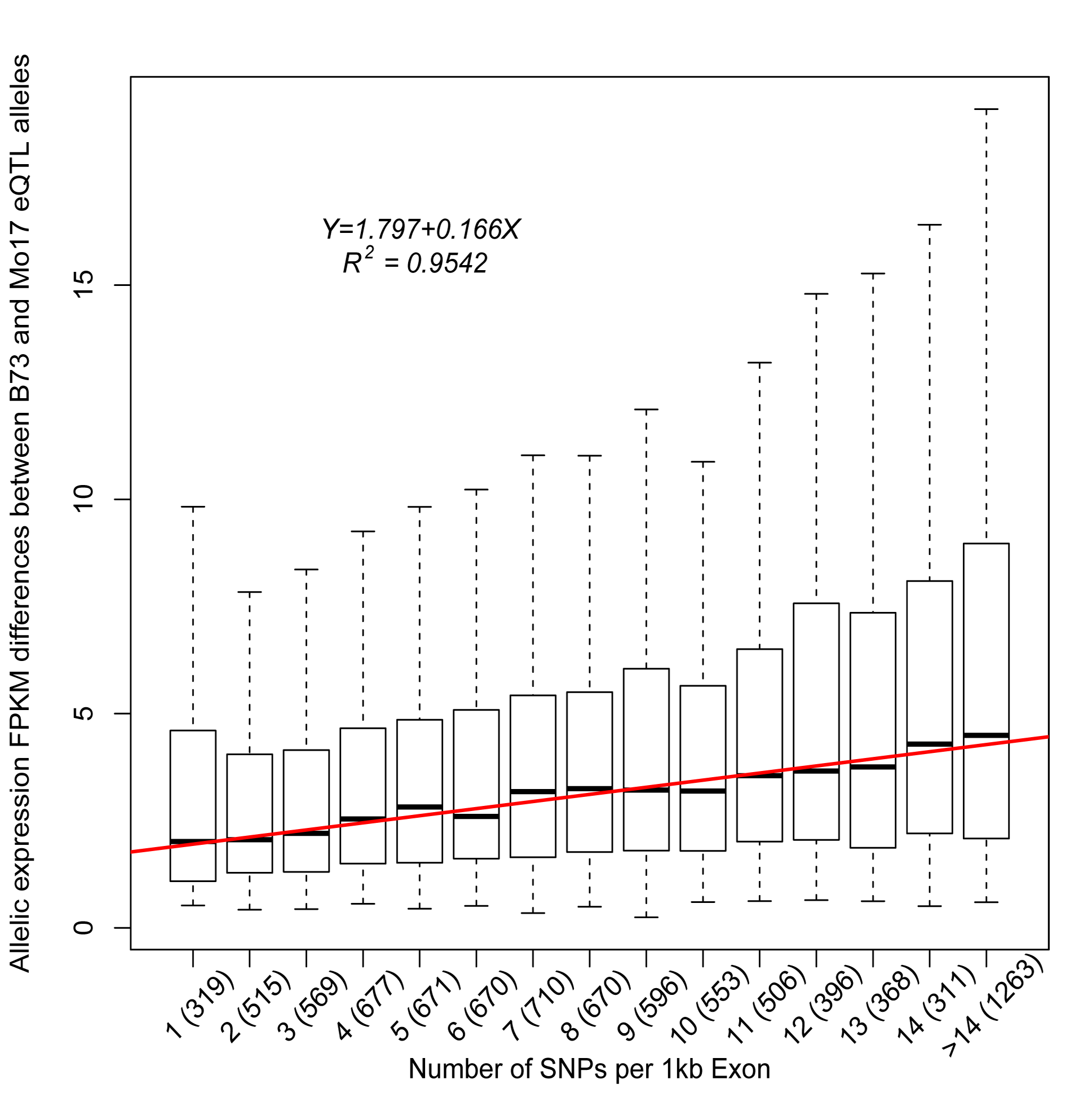

Supplement: Supplementary file 3 — Additional file 3: Fig. S3 For genes with B73 positive cis-eQTL when aligned to the B73 genome by TopHat2 with the default parameters, the expression level differences of homozygous B73 and Mo17 lines were plotted relative to SNP frequency. Exon sequence divergence was calculated the number of SNPs matching exons in a gene divided by the total exon lengths and multiplied by 1000. The numbers in parenthesis represent the numbers of genes in each group. Allelic expression differences between B73 and Mo17 were highly correlated with numbers of SNPs per 1 kb exon for the 8794 B73 positive cis-eQTL genes with SNP information. The y-axis is the allelic expression level difference of B73 minus Mo17 in FPKM, or two times the expected additive effect of the locus. [file 12864_2021_7577_MOESM3_ESM.tif]
